# Traumatic Shock

**Published:** 1919

**Authors:** W. B. Cannon

**Affiliations:** U. S. Army


### TRAUMATIC SHOCK

By Major W. B. Cannon, U. S. Army.

Abstract of discussion before the Société de Biologie, Oct. 19, 1918.

*Definition.* Traumatic shock is a general bodily state occurring after wounds and characterized by persistent low arterial pressure, rapid pulse, pallor, sweating, and superficial, rapid respiration.

*Facts of Observation.* 1. Persistent low arterial pressure. — This depression is the central and peculiar feature of shock and is a measure of its degree.

2. Concentration of corpuscles in capillaries. — The difference between capillary and venous erythrocyte counts in shock may be as much as 2,500,000 corpuscles per cubic millimetre (Béthune observations).

3. Concomitant variation of the degree of shock and body temperature. — The blood pressure falls as the shocked man becomes cold, and rises as he is warmed.

4. Reduction of the alkali reserve (i. e., of the sodium bicarbonate of the blood) in many cases of shock. — The reduction is often so great as to constitute acidosis in Van Slyke's definition (Béthune observations).

5. A correspondence between the degree of reduction of the alkali reserve and the degree of lowering of arterial pressure in shocked men (Béthune observations).

6. Damage to the circulatory control. — If a low pressure has lasted a long time, nothing will cause a permanent rise of blood pressure (Dijon experiments, clinical observations).

7. A marked sensitiveness of shocked men to ether or chloroform anesthesia, which induces a dangerous reduction of an already low pressure (Béthune observations and Dijon experiments).

8. A tolerance of nitrous oxide and oxygen as an anesthetic without fall of blood pressure (Béthune observations and Dijon experiments).

*Explanation of these Facts.* 1. Low arterial pressure. — This is not due to cardiac weakness; for if arterial pressure is raised by adrenalin or by cerebral compression, the heart works effectively against the high pressure (Mann, Dijon experiments). It is not due primarily to lack of vasoconstrictor tone; for, raising the blood pressure by aortic compression, in a rabbit with cervical sympathetic cut on one side, results in flushing of the denervated ear but not of the ear still connected with the medulla (Seelig and Joseph).

2. The low pressure is explicable as a consequence of blood being out of circulation or stagnant in some part of the vascular system (exæmia). The exæmic blood is not in the abdominal veins as commonly supposed (see testimony of British surgeons, *Lancet*, 1917, 2, p. 727). It is not in the arteries, for in that case arterial pressure would not be low.

3. Concentration of blood in capillaries. — The observation suggests that the "lost blood" of shock may be largely stagnant in capillaries, concentrated there by extravasation of lymph. Concentration is seen also in shock produced by injection of histamine (Dale and Laidlaw).

4. Variation of shock with body temperature. — Cold causes an accumulation and concentration of corpuscles in the capillaries. The blood count is higher in a cold than in a corresponding warm part of the skin. Cold, therefore, would augment the exæmia of shock and warmth would diminish it.

5. Reduction of the alkali reserve. — This results from too great lowering of arterial pressure. If the pressure is lowered to 80 mm. of mercury by lessening cardiac inflow through increased intrapericardial pressure, there is no reduction of the alkali reserve; if lowered to 30 mm. the reserve begins to decrease; and if lowered to 60 mm. it decreases more rapidly (Dijon experiments). Records of human cases made at Béthune confirm these experimental observations. A "critical level" in a falling blood pressure lies at about 80 mm. of mercury systolic pressure.

As arterial pressure falls, the circulation rate is slower and the oxygen supply to the tissues is less. Inadequate oxygen supply results in partial oxidation, with development of lactic acid (Araki), and with consequent reduction of the alkali reserve.

6. The lower the arterial pressure the lower the alkali reserve. From the foregoing considerations this result is to be expected.

7. Damage to the control of the circulation. — Nerve cells are specially sensitive to oxygen-lack. If blood pressure continues below the critical level, sensitive structures are injured. Vasoconstrictor tone gradually diminishes (Erlanger). Finally transfusion or infusion causes no permanent rise of pressure; the effect is that seen after the medulla is destroyed (Dijon experiments).

8. Sensitiveness to ether. — The injured vasoconstrictor centre is further depressed by the anesthetics, ether and chloroform, and blood pressure consequently drops to a lower level.

*The Cause of Shock.* 1. It is not due primarily to loss of vasoconstrictor tone, for reasons stated above.

2. It is not due to fat embolism; for, *a*) too much fat is required to check the flow of blood through the lungs (2 c. c. per kilo in dogs); *b*) venous pressure in man is not raised as in experimental fat embolism; *c*) the peculiar hyperpnœa or apnœa, seen in experimental fat embolism, does not occur in man.

3. It is not due to "acapnia". The excessive respiration demanded by this theory is not seen in human cases.

4. Evidence that it is due to the effect of tissue injury (experiments in co-operation with Bayliss and at Dijon; cf. Delbet, Quénu):

*a*) The crushing of muscles in a hind leg is followed by a fall of arterial pressure beginning in about 20 minutes and reaching in about an hour a shock level.

*b*) This effect occurs even though nerves to the leg are severed; it is therefore not of nervous origin.

*c*) If the blood vessels (iliac artery and vein) of the leg are tied and the muscle injured, the pressure drops only after the blood flow is restored.

*d*) If a shock pressure has been caused by muscle injury, tying the vessels may be followed by a progressive rise of pressure to the normal level.

*e*) The effects of muscle injury may pass away with spontaneous recovery of pressure.

*f*) The lowered pressure after injury is not primarily due to extravasation of blood and lymph into the injured tissues. The measured amount is insufficient.

*Application of the Foregoing Considerations to the Treatment of Shock.* 1. Every effort should be made to check loss of body heat from the shocked man and to restore to normal a lowered body temperature. Avoid exposing the body; use hot drinks, hot water bottles, blankets and hot air.

2. Raise as soon as possible an arterial pressure which persists below the critical level, in order to avoid the damage from oxygen want.

3. Use preferably transfusion of blood to raise pressure; for thus, in addition, oxygen carriers are added to the circulation. In the absence of blood, use Bayliss gum-salt solution, which raises pressure (by increasing volume) and thereby causes more rapid circulation and better employment of the corpuscles as oxygen carriers.

4. Use a tourniquet to separate a shattered, useless part from the rest of the body. Apply it as near as possible to the injured region, amputate proximal to the tourniquet and *before removing it*. If a tourniquet, used to stop bleeding, is to be left in place for a long period, apply it at the most distal effective location. The surgeon must be guided in his decision to save or sacrifice the part by questions of viability and gross infection in the excluded region, and danger to the rest of the body from its retention.

5. If ether is used in operating on a shocked man, begin to raise his blood pressure by transfusion or infusion as soon as the anesthetic is started, and continue the process during the operation. Use preferably nitrous oxide and oxygen, in a ratio not exceeding 3 : 1, preceded by morphia. Avoid always deep anesthesia and cyanosis.

(The Béthune observations were published in reports to the Sub-committee on Shock of the English Medical Research Committee, and in the Journal of the American Médical Association, February 23rd and March 2nd, 1918. The Dijon experiments have not been published in detail):

